# Trends and Characteristics of the US Adult Population’s Behavioral Patterns in Web-Based Prescription Filling: National Survey Study

**DOI:** 10.2196/23662

**Published:** 2021-03-16

**Authors:** Lin-Ya Yang, Jennifer G Lyons, Steven R Erickson, Chung-Hsuen Wu

**Affiliations:** 1 School of Pharmacy College of Pharmacy Taipei Medical University Taipei Taiwan; 2 Department of Population Medicine Harvard Medical School and Harvard Pilgrim Health Care Institute Boston, MA United States; 3 Department of Clinical Pharmacy College of Pharmacy University of Michigan Ann Arbor, MI United States

**Keywords:** internet, prescription, online pharmacy, National Health Interview Survey, trend

## Abstract

**Background:**

Filling a prescription on the web has become an alternative to in-person pharmacies for individuals to access their medications. However, the adoption of web-based filling has been gradual, and the use patterns remain to be unclear.

**Objective:**

This study aims to estimate the trend and prevalence of web-based prescription-filling behavior and identify associated factors among adults in the United States.

**Methods:**

We used data from the US National Health Interview Survey (NHIS) from 2009 to 2018. Adult respondents (aged ≥18 years and over) self-reported their behavior of web-based prescription filling, which was defined as having filled a prescription using the internet in the past 12 months during the survey year. We reported trends using weighted percentages adjusted by the NHIS complex sampling design. We used descriptive statistics and multivariable logistic regression models to examine trends and identify factors associated with web-based prescription-filling behavior.

**Results:**

The estimated number of adults reporting web-based prescription-filling behavior significantly increased from 13,319,877 (13,319,877/225,217,942, 5.91%) in 2009 to 28,308,262 (28,308,262/246,611,125, 11.48%) in 2018 *(P*<.001). Those who were more likely to report filling a prescription on the web were aged between 35 and 74 years, female, White, and frequent users of the computer or internet; these adults also reported higher education, higher income, insurance coverage, and poorer health status.

**Conclusions:**

Web-based prescription-filling behavior among US adults has increased significantly from 2009 to 2018. Health care providers should be aware of the upward trend in the use of web-based pharmacies and ensure the clinical safety of web-based prescriptions.

## Introduction

### Health Information and Internet

The widespread use of the internet as a means of obtaining health information and health care has a tremendous impact on health care systems [1,2]. It is estimated that more than 70% of internet users globally search for health information on the web [3-5]. The prevalence of web-based pharmacy use has increased over time [6,7]. Web-based pharmacies, which include web-based ordering and mail delivery, have become an alternative to obtaining prescription medications from in-person pharmacies. For example, more than 14% of internet users in the United States reported that they had ever bought medicines on the web [8]. According to data from the Health Information National Trends Survey (HINTS), the percentage of people in the United States who purchased medications on the web increased from 9.1% in 2003 to 20.2% in 2013 [7].

### Benefits and Concerns

Individuals may be increasingly interested in purchasing prescription medications on the web for several reasons. First, purchasing medication on the web is more convenient than an in-person pharmacy because web-based pharmacies are available 24 hours a day. People who have difficulty traveling to a physical pharmacy can more easily order medications on the web from home [9,10]. Second, purchasing on the web is more private and attractive for individuals who are concerned with obtaining their medications publicly from a community pharmacy [9,10]. Finally, given that the cost of prescription medication continues to increase in the United States [11], saving money is the leading reason for purchasing medication on the internet, although mostly from foreign websites [12,13].

Despite the perceived benefits, there remain several concerns regarding the use of web-based pharmacies to obtain prescriptions. Underregulated or illegal web-based pharmacies can sell medications that may contain the wrong dosage or active ingredients or be past the expiration date or are not approved by the US Food and Drug Administration [10,14]. The 2018 report of the National Association of Boards of Pharmacy (NABP) showed that among the 11,943 web-based pharmacies reviewed, the vast majority (95%) were found to be noncompliant with relevant federal or state pharmacy laws [15]. Furthermore, less than 3.0% of the reviewed web-based pharmacies were accredited by the NABP-verified internet pharmacy practice sites [15].

### Purchasing Medicines on the Web

Several previous studies have examined web-based purchasing medications [1,8,16-22]. These studies have explored the characteristics of purchasers using web-based pharmacies [1,8,16,18-22], the experience of purchasing medications on the web [16,17,19-21], reasons for purchasing medications on the web [19,20,22], and patients’ tendency to disclose to their health care providers that they purchase medications using web-based pharmacies [8]. However, most of the previous research has studied web-based medication-purchasing behaviors from other countries [16,17,19,20,22] but not specifically from the United States. Moreover, one difference between the United States and many other countries is that prescription-only medication can be purchased using web-based pharmacies that are located within the United States [16,17,19,20,22]. Rules vary by country, from very restrictive (no internet pharmacy allowed) to allowing the purchase of prescription medication from another country. For example, unlike the United States, some other countries allow only nonprescription medications to be purchased on the web [23]. Finally, the data obtained are typically from specific hospitals [17,21] or community pharmacies [16] or from mailed surveys of a specific sample [20,22], not using a nationally representative survey design. Only a few studies used nationally representative US data to investigate web-based pharmacy use [1,8,18]. For example, one study was conducted using the Medical Expenditure Panel Survey (MEPS) to evaluate characteristics associated with web-based pharmacy use [18], whereas another study was conducted using the HINTS to evaluate the characteristics of people purchasing medication on the web [8]. However, the findings from the MEPS and HINTS studies were published 10 years ago, and more updated evidence is needed to understand newer trends in the use of web-based pharmacies.

### Objective

With the expansion of internet use [24] and the recent COVID-19 pandemic [25], purchasing medications using web-based pharmacies may be the new normal for individuals to access their medications in the future. Therefore, it is important to update the information available regarding the prevalence and utilization patterns of people filling prescriptions using web-based pharmacies. Using data from the National Health Interview Survey (NHIS), which is a nationally representative data set of the United States, provides this unique opportunity. This study aims to (1) evaluate the trend and (2) identify the characteristics associated with web-based prescription-filling behavior among the US adult population.

## Methods

### Data Source

We used data from the US NHIS, from 2009 to 2018, which is a cross-sectional household interview survey designed to track health status, health care access, and health resource utilization among the civilian noninstitutionalized population in the United States. [26]. The NHIS was initiated in 1957 and is conducted by the National Center for Health Statistics (NCHS), Centers for Disease Control and Prevention (CDC), which releases the survey data annually [26].

The data in the NHIS were obtained through a complex sample design involving stratification, clustering, and weighting. Given the changes in the distribution of the US population, a new sample design was implemented in 2016 [27]. The data of the NHIS are collected from face-to-face interviews. Therefore, to keep the data collection more manageable and cost-effective, the sample was systematically selected using multistage sampling technologies (survey data before 2016 NHIS) [28] and geographically clustered sampling techniques (survey data from 2016 NHIS) [27]. Multistage sampling technologies partition the US population into several nested levels of strata and clusters consisting of a sample of primary sampling units (PSUs) [28]. Respondents were selected from the PSUs to form the sample. Regarding the geographically clustered sampling techniques, the sampling process started by dividing the United States into many geographic areas. One geographic area included 1 or 2 strata, and 1 stratum contained many clusters of addresses. Then, a specific number of clusters according to the size of the strata would be selected for the NHIS sample [26,27]. 

We used data from the Household File, Family File, Person File, and Sample Adult File from the NHIS for this study. From 2009 to 2018, the survey contained approximately 35,000 households, yielding about 87,500 respondents who participated in the survey each year [26]. The number of respondents can be weighted to obtain estimates of the US population. The response rates each year were 82.2%, 79.5%, 82.0%, 77.6%, 75.7%, 73.8%, 70.1%, 67.9%, 66.5%, and 64.2% [26].

In 2009, the NHIS started collecting data on the use of health information technology in the Sample Adult File. A total of 5-10 questions, depending on the year, about using the internet to search for health information, learn about health topics, fill a prescription, schedule medical appointments, and email health care providers were asked in a face-to-face interview [29]. The NHIS did not release these data in 2010. More detailed information about study design, interview procedure, data editing, and survey questionnaires is available on the official website.

### Study Population

All respondents aged ≥18 years who participated in the survey were included as the study population.

### Study Outcome (Web-Based Prescription-Filling Behavior)

The survey contained one question asking respondents if they had ever filled a prescription on the internet during the past 12 months. In 2009, the question was formatted as, “Did you refill a prescription on the internet in the past 12 months?” In the 2011 to 2018 surveys, the question was formatted as “DURING THE PAST 12 MONTHS, have you ever used computers for any of the following … Fill a prescription.” Respondents who answered “yes” to the question were defined as fillers of web-based prescription filling, and those who answered “no” were enrolled as nonfillers of web-based prescription filling.

### Covariates (Factors and Characteristics)

We used the Anderson Behavioral Model of Health Services Use as a conceptual framework to identify factors associated with the behavior of web-based prescription filling [30-32]. The Anderson model is widely used to study health behaviors and health service utilization [30-32]. According to the model, factors that are associated with health behavior or health services utilization can be classified into predisposing, enabling, and need factors. In this study, we identified all the variables from the Family Files, Person Files, and Sample Adult Files in the NHIS and further divided them into 3 categories based on the Anderson model.

Predisposing factors included age (range: 18-34, 35-49, 50-64, 65-74, and ≥75 years), gender (male and female), race (non-Hispanic White, Hispanic, non-Hispanic Black, non-Hispanic Asian, and non-Hispanic others), education (less than high school, high school, college, and higher than college), region (Northwest, Midwest, South, and West), marital status (married or living with partner, divorced or separated or widowed, and single or never married), work status (had job and no job), frequency of computer usage (never, some days, most days, and every day), and frequency of internet usage (never, per day, per week, per month, and per year). Enabling factors included family income (<US $35,000, US $35,000-49,999, US $45,000-74,999, US $75,000-$99,999, and ≥US $100,000) and health insurance coverage (covered and not covered). Needs factors included perceived health status (excellent, very good, good, fair, and poor), mobility limitations (yes or no), prescribed medication by doctors or health professionals (yes or no), and drugs bought from another country to save money (yes or no). It should be noted that some variables, including frequency of computer usage, frequency of internet usage, prescribed medication by doctors or health professionals, and drugs bought from another country to save money, were only recorded in the 2018 survey but not in the 2009 survey. Therefore, these variables can only be analyzed using the 2018 survey data.

### Statistical Analysis

A total of 3 major statistical analyses were performed in our study. First, we estimated the annual weighted percentage of web-based prescription-filling behavior among US adults from 2009 to 2018. To evaluate the trend change in web-based prescription-filling behavior, we compared the prevalence between 2009 and 2018. In addition to the overall trend of web-based prescription-filling behavior, we also examined the trends stratified by demographic subpopulation, including age, gender, race, and education levels. Second, we compared selected factors among respondents in 2009 and 2018 separately, using descriptive statistics to identify changes in patterns of web-based prescription-filling behavior. Wald chi-square tests were used to compare the categorized characteristics between web-based prescription fillers and nonfillers. Finally, we conducted multivariable logistic regression to evaluate the associations between factors and web-based prescription-filling behavior among the study population in 2018.

The NHIS uses a complex sample design (stratification, clustering, and weighting) to represent the US population. Sampling weights are necessary for numbers from respondents to produce representative national estimates [26]. The weight is the person-level base weight, which is adjusted according to poststratification and calibration by age, sex, race, and ethnicity classes based on the 2000 US census for survey in 2009 to 2015 and the 2010 census for survey in 2016 to 2018 [26,28].

All statistical procedures were performed using SAS software (version 9.4; SAS Institute). The SAS survey procedure (proc surveymean, proc surveyfreq, and proc surveylogistic) was used to perform the analyses after considering the stratification, clustering, and weighting of the NHIS sample design to represent the US population and prevent underestimation of variances [33]. The two-sided test was used, and statistical significance was set at *P*<.05. As the NHIS data are publicly available and deidentified, this study was exempt from a full ethical review by the Joint Institutional Review Board of Taipei Medical University.

## Results

### Trend and Prevalence of Web-Based Prescription Fill

**Figure 1:**
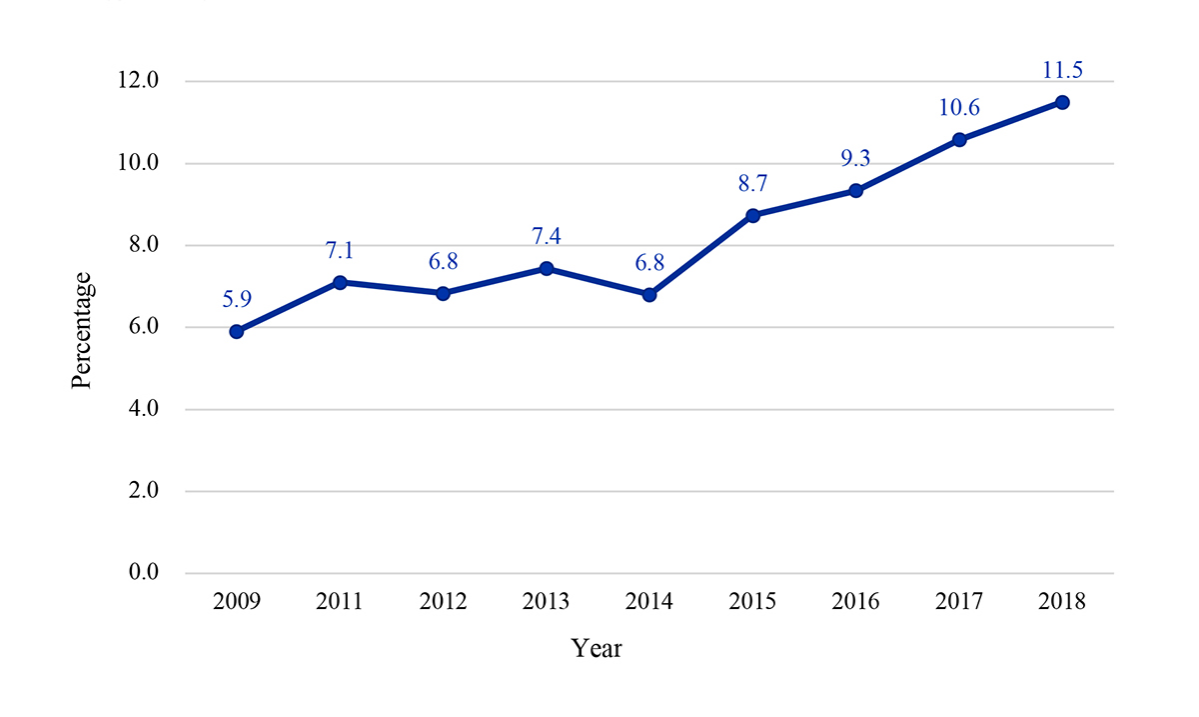
Trends in web-based prescription-filling behavior among US adults from 2009 to 2018. The prevalence of online prescription fill among adults in the US was found to significantly increase from 2009 (N=13,319,877) to 2018 (N=28,308,262; *P*<.01). The National Health Interview Survey did not release the data related to online prescription fill in the 2010 survey.

Figure 1 shows the trend of web-based prescription fill from 2009 to 2018 among adults in the United States. All the numbers were weighted to obtain US population estimates. The estimated number of respondents in the United States filling the prescription on the web significantly grew from 13.3 million in 2009 to 28.3 million in 2018 (*P*<.001). The overall annual prevalence of web-based prescription filling was relatively constant between 2009 (13,319,877/225,217,942, 5.91%) and 2014 (16,072,655/236,126,315, 6.81%) but increased from 2014 (16,072,655/236,126,315, 6.81%) to 2018 (28,308,262/246,611,125, 11.48%).

**Figure 2:**
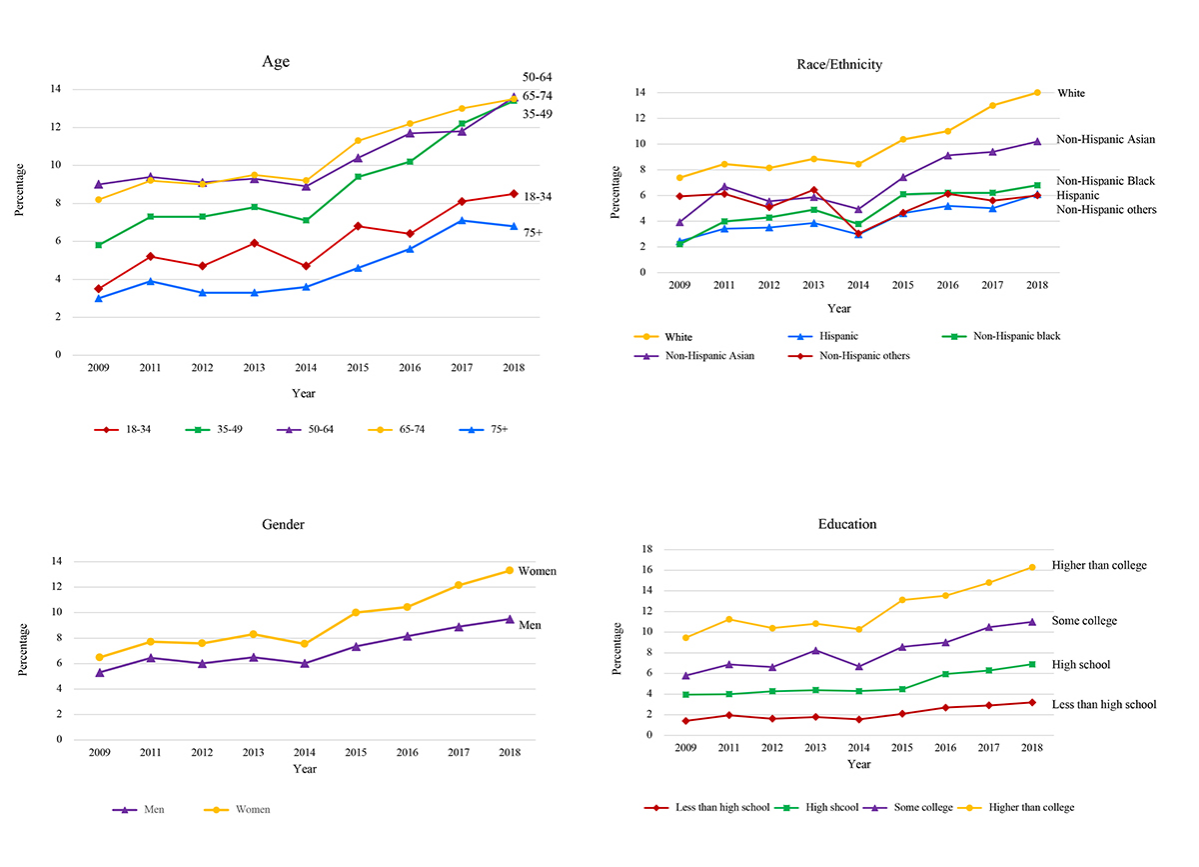
Prevalence of web-based prescription-filling behaviors among adults in the United States by demographic variables, 2009 to 2018.

The trends for demographic subpopulations are shown in Figure 2. In subpopulations, trends in the prevalence of web-based prescription-filling behavior were similar to the overall trend in the general adult population. In the trend of web-based prescription-filling behavior by age, respondents aged between 18 and 24 years and ≥75 years were the least likely to fill a prescription on the internet. In contrast, respondents aged 35-74 years were more likely to fill prescriptions on the web, and the percentage significantly increased during the past decade (*P*<.001).

In terms of gender differences, the prevalence of web-based prescription-filling behavior was consistently greater in women than in men from 2009 to 2018. In both genders, the behavior of web-based prescription filling significantly increased from 2009 to 2018 (*P*<.001). Among all ethnic groups, non-Hispanic White respondents were most likely to fill the prescription on the web, followed by non-Hispanic Asian respondents (*P*<.001). The higher the education level, the more likely adults would choose to fill a prescription on the web (*P*<.001). The prevalence of web-based prescription-filling behavior for respondents with a higher than college degree was consistently the highest among adult respondents in the United States. In contrast, adults with an education level less than high school were less likely to fill a prescription on the web, and the percentage of use remained consistently low from 2009 to 2018.

### Characteristics of Study Populations

Table 1 shows the characteristics of the 2009 and 2018 study populations. In both 2009 and 2018, several characteristics, such as age (*P*<.001), sex (*P*=.003 in 2009 and *P*<.001 in 2018), race or ethnicity (*P*<.001), education (*P*<.001), marital status (*P*<.001), work status (*P*<.001), family income (*P*<.001), and health insurance coverage (*P*<.001), were associated with a higher probability of web-based prescription-filling behavior. Moreover, variables including frequency of computer usage (*P*<.001), frequency of internet usage (*P*<.001), prescribed medication by doctors or health professionals (*P*<.001), and having bought drugs from another country to save money (*P*<.001) were found to be significantly associated with web-based prescription-filling behavior in 2018.

Within each characteristic, respondents aged between 50 and 64 years, female, White, and who had higher education, higher income, and insurance coverage were significantly more likely to fill prescriptions on the web in both 2009 and 2018. In addition, respondents who were married or lived with a partner were significantly more likely to fill a prescription on the web when compared with those who were divorced or widowed or single or never married (10,012,769/138,405,448, 7.23% vs 1,659,300/38,520,573, 4.31% vs 1,632,904/48,049,055, 3.40%; *P*<.001 in 2009; 19,956,831/147,945,646, 13.50% vs 3,757,131/40,811,170, 9.21% and 4,584,495/57,461,184, 8.00%; *P*<.001 in 2018).

In terms of working status, respondents who had a job in the past 12 months were more likely to complete the prescription on the web than respondents without a job (10,172,125/157,802,261, 6.4% vs 3,147,752/67,277,067, 4.7%; *P*<.001 in 2009; 20,794,336/169,686,451, 12.3% vs 7,486,229/76,519,060, 9.8%; *P*<.001 in 2018). Perceived health status and region were significantly associated with web-based prescription filling in 2018 (*P*<.001 and *P*<.001, respectively) but not in 2009. In 2018, the region was significantly different, with people in the Midwest and West having a higher filling behavior compared with people in the Northwest or the South.

**Table 1:** Characteristics and a comparison between web-based prescription fillers and nonfillers in the past 12 months during the survey year: weighted statistics in 2009 (n=1489; N=13,319,877)^a^ and 2018 (n=2892; N=28,308,262)^a^.

Variables			2009			2018		
			Yes	No	*P* value	Yes	No	*P* value
**Predisposing factors, weighted n (%)**								
	**Age (years)**				<.001			<.001
		18-34	2,424,539 (3.52)	66,471,375 (96.48)		6,200,449 (8.49)	66,865,684 (91.51)	
		35-49	3,643,127 (5.80)	59,175,765 (94.20)		8,149,953 (13.44)	52,483,963 (86.56)	
		50-64	5,070,551 (9.03)	51,100,111 (90.97)		8,440,377 (13.62)	53,550,362 (86.38)	
		65-74	1,668,375 (8.18)	18,729,467 (91.82)		4,126,912 (13.53)	26,381,215 (86.47)	
		75+	513,285 (3.03)	16,421,347 (96.97)		1,390,571 (6.81)	19,021,639 (93.19)	
	**Gender**				.003			<.001
		Male	5,771,128 (5.31)	102,970,784 (94.69)		11,324,986 (9.51)	107,766,068 (90.49)	
		Female	7,548,749 (6.48)	108,927,281 (93.52)		16,983,276 (13.32)	110,536,795 (86.68)	
	**Race**				<.001			<.001
		Non-Hispanic White	11,436,419 (7.38)	143,504,508 (92.62)		22,072,419 (14.01)	135,502,059 (85.99)	
		Hispanic	759,766 (2.44)	30,349,873 (97.56)		2,444,137 (6.05)	37,945,521 (93.95)	
		Non-Hispanic Black	589,266 (2.21)	26,039,713 (97.79)		2,033,915 (6.76)	28,056,833 (93.24)	
		Non-Hispanic Asian	411,558 (3.93)	10,057,804 (96.07)		1,565,100 (10.20)	13,773,476 (89.90)	
		Non-Hispanic others	122,868 (5.94)	1,946,167 (94.06)		192,691 (5.99)	3,024,974 (84.01)	
	**Education**				<.001			<.001
		Less than high school	563,916 (1.41)	39,347,807 (98.59)		1,098,243 (3.19)	33,369,627 (96.81)	
		High school	2,189,184 (3.95)	53,292,533 (96.05)		3,721,460 (6.94)	49,863,572 (93.06)	
		College	2,621,675 (5.80)	42,561,073 (94.20)		5,036,039 (10.96)	40,928,096 (89.04)	
		Higher than college	7,407,794 (9.47)	70,847,409 (90.53)		16,952,950 (16.35)	86,765,573 (83.65)	
	**Region**				.43			<.001
		Northwest	2,200,012 (5.61)	37,027,777 (94.39)		4,128,255 (9.69)	38,483,459 (90.31)	
		Midwest	3,250,003 (5.96)	51,305,184 (94.04)		7,115,471 (13.10)	47,180,604 (86.90)	
		South	4,566,581 (5.67)	76,000,333 (94.33)		9,709,220 (10.67)	81,307,356 (89.33)	
		West	3,303,281 (6.49)	47,564,771 (93.51)		7,355,316 (12.53)	51,331,444 (87.47)	
	**Marital status**				<.001			<.001
		Married or living with partner	10,012,769 (7.23)	128,392,679 (92.77)		19,956,831 (13.49)	127,988,815 (86.51)	
		Divorced, separated, widowed	1,659,300 (4.31)	36,861,273 (95.69)		3,757,131 (9.21)	37,054,039 (90.79)	
		Single, never married	1,632,904 (3.40)	46,416,151 (96.60)		4,584,495 (7.98)	52,876,689 (92.02)	
	**Work status (past 12 months)**				<.001			<.001
		Had job	10,172,125 (6.45)	147,630,136 (93.55)		20,794,336 (12.25)	148,892,115 (87.75)	
		No job	3,147,752 (4.68)	64,129,315 (95.32)		7,486,229 (9.78)	69,032,831 (90.02)	
	**Frequency of computer usage**				—^b^			<.001
		Never	—	—		613,413 (1.31)	46,114,456 (98.71)	
		Some days	—	—		1,248,875 (4.84)	24,531,551 (95.16)	
		Most days	—	—		1,587,924 (11.62)	12,072,018 (88.38)	
		Every day	—	—		24,802,396 (15.54)	134,817,997 (84.46)	
	**Frequency of internet usage**				—			<.001
		Never	—	—		565,220 (1.29)	43,402,368 (98.71)	
		Per day	—	—		22,391,668 (14.56)	131,380,588 (85.44)	
		Per week	—	—		4,275,620 (11.22)	33,817,721 (88.78)	
		Per month	—	—		61,283 (2.47)	2,415,606 (97.53)	
		Per year	—	—		260,601 (15.30)	1,442,600 (84.70)	
**Enabling factors,** **weighted n (%)**								
	**Family income (US $)**				<.001			<.001
		<35,000	1,752,244 (2.51)	68,111,146 (97.49)		3,354,653 (5.79)	54,557,513 (94.21)	
		35,000-49,999	1,494,075 (4.91)	28,937,754 (95.09)		2,301,726 (9.35)	22,311,049 (90.65)	
		45,000-74,999	2,476,437 (6.20)	37,486,119 (93.80)		4,179,405 (11.07)	33,558,936 (88.93)	
		75,000-99,999	2,138,734 (8.22)	23,884,329 (91.78)		4,383,932 (14.32)	26,227,420 (85.68)	
		≥100,000	4,749,518 (10.66)	39,788,717 (89.34)		12,334,689 (17.02)	60,133,071 (82.98)	
	**Health insurance coverage**				<.001			<.001
		Covered	12,790,369 (6.93)	171,671,557 (93.07)		27,309,470 (12.42)	192,643,180 (87.58)	
		Not covered	516,217 (1.29)	39,527,626 (98.71)		927,280 (3.65)	24,489,010 (96.35)	
**Needs factors** **, weighted n (%)**								
	**Perceived health status**				.25			<.001
		Excellent	3,682,424 (5.74)	60,522,896 (94.26)		6,476,485 (9.54)	61,394,808 (90.46)	
		Very good	4,582,321 (6.38)	67,271,787 (93.62)		10,517,116 (12.88)	71,134,469 (87.12)	
		Good	3,539,990 (5.90)	56,413,051 (94.10)		7,737,178 (11.77)	57,995,479 (88.23)	
		Fair	1,190,494 (5.46)	20,595,996 (94.54)		2,864,476 (11.81)	21,392,785 (88.19)	
		Poor	324,648 (4.41)	7,033,584 (95.59)		713,007 (10.20)	6,278,595 (89.80)	
	**Mobility limitations**				.07			.20
		Yes	565,452 (4.55)	11,866,412 (95.45)		1,645,744 (10.36)	14,233,084 (89.64)	
		No	12,754,425 (5.99)	200,021,362 (94.01)		26,662,518 (11.56)	204,004,900 (88.44)	
	**Prescribed medication by doctor** or **health professional**				—			<.001
		Yes	—	—		25,516,673 (15.77)	136,339,307 (84.23)	
		No	—	—		2,777,904 (3.29)	81,750,890 (96.71)	
	**Bought drugs from another country to save money**				—			<.001
		Yes	—	—		745,833 (19.10)	3,159,455 (80.90)	
		No	—	—		27,555,721 (11.36)	215,095,537 (88.64)	

^a^The “n” here is the number of the respondents, and the “N” here is the weighted population of the respondents.

^b^The following variables were not recorded in the 2009 survey: frequency of computer usage, frequency of internet usage, prescribed medication by doctors or health professionals, and drugs bought from another country to save money.

### Results From the Multivariable Regression Model

Table 2 shows the results of a multivariable logistic regression model to evaluate the association between respondent characteristics and web-based prescription-filling behavior among respondents in 2018. After adjustment, respondents aged between 35 and 74 years, female, higher education level, and with health insurance coverage were significantly associated with web-based prescription-filling behavior. In this adjusted model, Hispanic and non-Hispanic Black individuals were found to be significantly less likely to fill the prescription on the web than non-Hispanic White individuals. Persons living in the Midwest, South, and West were more likely to fill prescriptions using web-based pharmacies than those living in the Northwest of the United States.

**Table 2:** Factors associated with web-based prescription-filling behavior among adults in the United States in 2018: results from the adjusted multivariable logistic regression model.

Variables			Year (2018), odds ratio (95% CI)	
**Predisposing factors**				
	**Age (years)**			
		18-34		Ref^a^
		35-49		1.27 (1.08-1.50)
		50-64		1.22 (1.03-1.44)
		65-74		1.38 (1.13-1.68)
		75+		0.89 (0.68-1.17)
	**Gender**			
		Male		Ref
		Female		1.38 (1.23-1.55)
	**Race**			
		Non-Hispanic White		Ref
		Hispanic		0.71 (0.57-0.89)
		Non-Hispanic Black		0.72 (0.56-0.91)
		Non-Hispanic Asian		0.83 (0.63-1.09)
		Non-Hispanic others		0.69 (0.36-1.30)
	**Education**			
		Less than high school		Ref
		High school		1.40 (1.04-1.89)
		College		1.64 (1.21-2.21)
		Higher than college		2.26 (1.70-3.01)
	**Region**			
		Northwest		Ref
		Midwest		1.51 (1.24-1.84)
		South		1.30 (1.09-1.56)
		West		1.67 (1.36-2.04)
	**Marital status**			
		Married or living with partner		Ref
		Divorced, separated, widowed		0.86 (0.74-1.00)
		Single, never married		0.96 (0.81-1.14)
	**Work status (past 12 months)**			
		Had job		1.00 (0.87-1.15)
		No job		Ref
	**Frequency of computer usage**			
		Never		Ref
		Some days		1.62 (1.06-2.50)
		Most days		2.65 (1.63-4.33)
		Every day		3.65 (2.38-5.62)
	**Frequency of internet usage**			
		Never		Ref
		Per day		3.48 (2.11-5.73)
		Per week		3.24 (1.97-5.34)
		Per month		1.20 (0.41-3.52)
		Per year		3.26 (1.45-7.35)
**Enabling factors**				
	**Family income (US $)**			
		<35,000		0.78 (0.65-0.93)
		35,000-49,999		1.02 (0.84-1.23)
		45,000-74,999		Ref
		75,000-99,999		1.12 (0.94-1.35)
		≥100,000		1.26 (1.08-1.48)
	**Health insurance coverage**			
		Covered		1.60 (1.19-2.14)
		Not covered		Ref
**Needs factors**				
	**Perceived health status**			
		Excellent		Ref
		Very good		1.26 (1.09-1.46)
		Good		1.39 (1.19-1.63)
		Fair		1.80 (1.44-2.24)
		Poor		1.81 (1.27-2.58)
	**Mobility limitations**			
		Yes		1.23 (0.96-1.58)
		No		Ref
	**Prescribed medication by doctor** or **health professional**			
		Yes		4.74 (3.83-5.87)
		No		Ref
	**Bought drugs from another country to save money**			
		Yes		2.10 (1.51-2.93)
		No		Ref

^a^Ref: reference value.

Furthermore, the frequency of computer usage was also a significant predictor of web-based prescription-filling behavior. For example, respondents who used a computer for some days, most days, and every day were all significantly more likely to report that they filled a prescription on the web. Compared with people with excellent health status, individuals with very good, good, fair, and poor health status were 1.26, 1.39, 1.80, and 1.81 times more likely to fill the prescription on the web, respectively. In addition, the probability of filling the prescription on the web was 2.10 times greater in people who had bought drugs from another country to save money. Finally, unlike the results of the unadjusted analysis, marital status and work status were not found to be significantly associated with web-based prescription-filling behavior after adjustment.

## Discussion

### Principal Findings

To our knowledge, this is the most recent study to investigate the trends and patterns of web-based prescription-filling behavior among a nationally representative population of adults in the United States. The results showed that the prevalence of web-based prescription filling has significantly increased from 2009 to 2018. Individuals who filled a prescription on the web were more often between the ages of 35 and 74 years, female, White, of higher education, used the computer or internet frequently, had insurance coverage, and had poorer health status.

The findings from the survey of HINTS showed that the trend of obtaining medications or vitamins on the web increased from 2003 to 2013 [7]. Our study supported the findings from the HINTS survey and provided updated results of web-based prescription-filling behavior among adults in the United States.

In our study, characteristics including age, gender, race, education, income, and insurance coverage were found to be associated with filling prescriptions using web-based pharmacies. With regard to age, when compared with people aged between 18 and 34 years, people aged between 35 and 74 years were significantly more likely to fill a prescription on the web. The oldest group, those aged 75 years and older, was the least likely to fill a prescription on the web. There is a steady decrease in the prevalence of internet use by age. A previous study that investigated internet use among older adults showed that 82% of seniors aged 65-69 years used the internet, and the prevalence diminished with age with 60% of those aged 65-79 years and 44% of those aged ≥80 years saying they used the internet [34]. It is not surprising that older adults were least likely to fill their prescriptions on the web compared with other age groups, as these individuals usually have difficulties in adapting to technology, which makes web-based prescription filling less likely to become an alternative to obtaining prescription medications in person.

Gender is a strong predictor of web-based prescription-filling behavior. We found that the prevalence of web-based prescription-filling behavior was consistently higher in women than in men from 2009 to 2018. This may be because of the tendency of females to adapt to behaviors of web-based health technology use [35-39] and partially explained by the gender difference in health perceptions and health service utilization [35-39].

Those with higher income were also found to be more likely to fill a prescription on the web. Higher income enables people to access the computer and internet more easily, which makes them more capable of filling prescriptions on the web. Insurance coverage was also found to be associated with a higher likelihood of web-based prescription filling. A survey conducted in 1797 web-based pharmacy users and their reasons for use found that people were encouraged or even compelled to use mail-order pharmacies because of the requirement of certain health insurance plans [21]. This might explain why health insurance coverage was significantly associated with web-based prescription filling in this study.

Educational status was found to be associated with web-based prescription-filling behaviors. People with higher education were significantly more likely to fill prescriptions on the web. People with higher education often have higher health literacy, which in turn may help them access the internet more easily [40,41]. Better health literacy with higher education enables these people to understand health information easily and choose an alternative and convenient way to obtain prescriptions. In addition, higher education is linked to higher income and increases the likelihood of having a job that provides health insurance with a high-deductible insurance plan to encourage beneficiaries to fill a prescription on the web.

People with poor health status were found to be more likely to fill a prescription on the web. Poor health status could potentially result from having multiple health conditions. These individuals may have many prescriptions, which increases the complexity of the medication treatment. Web-based prescription-filling behavior provides the convenience of pharmacy access, which may explain the association between poor health status and a greater likelihood of filling a prescription using a web-based pharmacy. Previous research shows a varied relationship between health status and web-based pharmacy use. A greater burden of illness, as measured by the Charlson comorbidity index, was associated with a greater likelihood of using a web-based pharmacy [8,18], whereas another study using a health status scale similar to the one used in this study found no association [8]. Future research is needed to further understand the association between perceived health status and the behavior of web-based medication filling.

We also found that people who bought medications from another country to save money were twice as likely to fill their prescriptions on the web when compared with people who reported that they never bought medication from another country to save money. Our findings were consistent with a study from the Canadian International Pharmacy Association survey, which reported that saving money was the main reason that people purchased medications on the web, and the purchases were mostly from foreign websites [13]. Continuously rising prescription medication costs in the United States is one reason for the use of web-based pharmacies [11].

### Clinical Implications

From a clinical perspective, the safety of web-based prescriptions remains to be a major concern. A significant number of unqualified and illegitimate pharmacies are active on the web [14]. Furthermore, it is difficult to distinguish between a legal and illegal web-based pharmacy, which makes the safety of purchasing medications on the web even more of an issue. In addition, a previous study showed that few web-based pharmacy users disclose their use to health care providers [8]. One important reason might be that health care providers did not ask their patients about the experience of web-based pharmacy use [42]. The findings from this study confirm the need for health care providers to be aware of the increased and prevalent use of web-based prescription-filling behavior. In addition, health care providers should actively inquire, discuss, and educate their patients on ways to identify legal and qualified web-based pharmacies [8,17,43]. The advantages and convenience of the internet will make web-based pharmacy use even more prevalent in the near future.

Web-based prescription filling may be more in demand during the current COVID-19 pandemic and future crises. Epidemic prevention policies, such as quarantines and social distancing, can make the traditional way of filling prescriptions difficult or even impossible [25]. For example, a reduction in access to traditional pharmacies may occur because of pharmacy closures or quarantines of pharmacy staff, which could drive people to seek an alternative and convenient way, such as web-based pharmacies, to fill their prescriptions. Furthermore, different patient groups may be disproportionately impacted. On the basis of our findings, older people, minority people, and people with low income and education were less likely to fill a prescription on the web. Future studies should focus on further determining the limitations of accessing web-based pharmacies and developing appropriate interventions. The use of web-based prescription filling with mail order or home delivery among the underserved population during the pandemic crises may be one answer.

### Strengths

The strengths of this study include the use of data from the NHIS, which included a large, nationally representative sample of the US noninstitutionalized population. This database allowed us to update the trend of web-based prescription filling for a decade (2009-2018) and to identify characteristics associated with web-based prescription filling in a large cohort.

### Limitations

However, our study has several potential limitations. First, because the NHIS is a self-reported survey of respondents, recall bias can exist. Second, the NHIS asked respondents if they filled a prescription on the web but did not gather detailed information on the medication filled in this manner. There was no information about the class and type of medicine as well as reasons for filling a prescription on the web. Future research detailing the specific medications filled using web-based pharmacies, including prescription and nonprescription products, is warranted to further characterize patients’ use of web-based pharmacies. Third, the medication distribution channels may be disrupted during the pandemic. Patients with certain medication needs, such as patients enrolled in narcotic treatment programs who need methadone may have difficulty in maintaining their treatment. Although filling methadone using web-based sources is not an option at this time, further research is warranted to evaluate the implications of the pandemic on the drug dispensing and delivery system among certain groups of patients. Fourth, due to the nature of the survey data set and study design, only the association between predictor and outcome variables can be made. Fifth, we did not investigate the implication of disease condition on the web-based prescription-filling behavior because the NHIS has only selected disease states and not all disease states. However, disease condition and severity can affect the behavior of filling a prescription on the web. An individual with a certain disease condition may be more likely to fill a prescription on the web than an individual with a different disease condition. Future research needs to investigate the association between web-based prescription-filling behavior and disease condition or severity by collecting primary data. Finally, confounding factors may exist, specifically regarding insurance coverage. For example, a patient’s likelihood of filling a prescription on the web can result from his or her insurance coverage having lower copays or out-of-pocket expenses when prescriptions are filled on the web.

### Conclusions

In conclusion, our findings indicate that the prevalence of web-based prescription-filling behavior among US adults has increased significantly from 2009 to 2018. Adults in the United States who were aged between 35 and 74 years, were female, were White, had higher education, were frequent users of the computer or internet, and had higher income, insurance coverage, and poorer health status were significantly more likely to fill a prescription on the web. Health care providers should be aware of the increased use of web-based pharmacies to fill prescriptions and help educate patients about the appropriate use of legitimate web-based pharmacies.

## Abbreviations

CDC: Centers for Disease Control and Prevention
HINTS: Health Information National Trends Survey
ISPE: International Society for Pharmacoepidemiology
MEPS: Medical Expenditure Panel Survey
NABP: National Association of Boards of Pharmacy
NCHS: National Center for Health Statistics
NHIS: National Health Interview Survey
PSU: primary sampling unit
